# Short- and medium-term impact of bariatric surgery on the activities of CYP2D6, CYP3A4, CYP2C9, and CYP1A2 in morbid obesity

**DOI:** 10.1038/s41598-019-57002-9

**Published:** 2019-12-31

**Authors:** Jose Rodríguez-Morató, Albert Goday, Klaus Langohr, Mitona Pujadas, Ester Civit, Clara Pérez-Mañá, Esther Papaseit, Jose Manuel Ramon, David Benaiges, Olga Castañer, Magí Farré, Rafael de la Torre

**Affiliations:** 1 Department of Experimental and Health Sciences, Universitat Pompeu Fabra (CEXS-UPF), Dr. Aiguader 80, Barcelona, 08003 Spain; 2 Integrative Pharmacology and Systems Neuroscience Research Group, Neurosciences Research Program. IMIM (Hospital del Mar Medical Research Institute), Dr. Aiguader 88, Barcelona, 08003 Spain; 3 Spanish Biomedical Research Centre in Physiopathology of Obesity and Nutrition (CIBEROBN), Instituto Salud Carlos III, 28029 Madrid, Spain; 4 Department of Endocrinology and Nutrition, Hospital del Mar, Barcelona, Spain; 5 Department of Medicine, Autonomous University of Barcelona, Cerdanyola, Spain; 6 Cardiovascular Risk and Nutrition Research Group, IMIM (Hospital del Mar Medical Research Institute), Dr. Aiguader 88, Barcelona, 08003 Spain; 7 Department of Statistics and Operations Research, Polytechnic University of Catalonia, Barcelona, Spain; 8 Department of Pharmacology, Therapeutics and Toxicology, Autonomous University of Barcelona, Cerdanyola, Spain; 9 Clinical Pharmacology Unit, Hospital Universitari Germans Trias i Pujol (HUGTP-IGTP), Badalona, Spain; 10 Section of Gastrointestinal Surgery, Hospital del Mar, Barcelona, Spain

**Keywords:** Obesity, Phase II trials

## Abstract

Morbid obesity and bariatric surgery induce anatomical, physiological and metabolic alterations that may alter the body’s disposition of drugs. Current literature on this topic is limited and sometimes inconsistent. Cytochrome P450 (CYP) is a superfamily of enzymes that metabolize around 75% of all marketed drugs. The purpose of this study was to evaluate the impact of body mass index and bariatric surgery on CYP activities. Firstly, we evaluated the *in vivo* activity of 4 major CYP isoenzymes (CYP2D6, CYP3A4, CYP2C9, and CYP1A2) in normal weight, overweight, and morbidly obese individuals. Secondly, we assessed the short- (1 month) and medium-term (6 month) effects of the most commonly employed bariatric surgery techniques (laparoscopic sleeve gastrectomy and Roux-en-Y gastric bypass) on the activity of these enzymes. CYP3A4 activity was lower in morbidly obese individuals, compared to normal-weight controls. Interestingly, bariatric surgery normalized CYP3A4 activity. In comparison with normal-weight controls, morbidly obese individuals had higher CYP2D6 activity, which was only observed in individuals with two functional alleles for this isoenzyme. Neither body mass index nor surgery had significant effects on CYP2C9 and CYP1A2 activities. Overall, no relevant differences in CYP activities were found between surgical techniques. In conclusion, further studies should evaluate whether the observed alterations in CYP3A4 activity will require dose adjustments for CYP3A4 substrates especially in morbidly obese individuals before and after bariatric surgery.

## Introduction

The prevalence of obesity has increased dramatically over recent last decades. According to the WHO, 39% of adults aged 18 years and over were overweight in 2016, and 13% were obese^1^. In severe obesity, life expectancy is reduced by an estimated 5 to 20 years, particularly among younger adults^2,3^. Morbid obesity is often associated with several pathologies including type 2 diabetes, hypertension, dyslipidemia, coronary artery disease, asthma, obstructive sleep apnea, and osteoarthritis^4^. Consequently, morbidly obese patients typically require pharmacological treatment to deal with such conditions.

Both obesity^5^ and bariatric surgery^6^ induce anatomical, physiological, and metabolic alterations that may affect the pharmacokinetics (including absorption, distribution, metabolism, and excretion) of drugs. This is especially relevant in drugs with a narrow therapeutic index since the required dose could be modified to such an extent that the drug becomes inactive (by being under the minimum effective concentration) or toxic (by being over the maximum safe one)^7,8^.

Currently, there is a lack of knowledge regarding how the bioavailability and metabolism of drugs are altered in morbid obesity and/or after bariatric surgery^9^, moreover, the information available is limited^10^ and sometimes inconsistent^5^. As a consequence, there are only a few, specific recommendations with respect to the pharmacological dose in patients who have undergone bariatric surgery^11^, and dose selection guidelines are rarely reported in reference textbooks and product package inserts^12,13^.

Cytochrome P450 (CYP) is a superfamily of heme-containing enzymes which metabolize most xenobiotics, including around 75% of all marketed drugs^14^. These enzymes catalyze the majority of phase I biotransformation reactions (reduction, oxidation, and hydrolysis) which generally lead to more polar compounds^15^. CYP genes are highly polymorphic, and variability in CYP content and activities have a considerable impact on drug pharmacokinetics and clinical response^16^. Genetic polymorphisms of CYP are of particular relevance for CYP2A6, CYP2C9, CYP2C19, and CYP2D6 and lead to different pharmacogenetic phenotypes (e.g. poor, intermediate, extensive, and ultrarapid metabolizers)^15,17^.

Both morbid obesity and bariatric surgery, together with the subsequent changes in weight and body composition, are key parameters that could affect CYP activities and, consequently, the metabolism of various drugs^14,18–20^. Although there are a number of reports evaluating the effect of obesity on the activity of some CYP isoenzymes (e.g. CYP3A4, CYP1A2, CYP2C9, CYP2C19, and CYP2D6) most of them, as previously stated, are inconsistent and do not allow conclusions to be drawn^5^. To the best of our knowledge, no study has yet evaluated in depth the consequences of bariatric surgery on the activity of multiple CYP isoenzymes. In a previous report, we described how paracetamol pharmacokinetics and caffeine metabolism were altered after bariatric surgery^21^. In the present study, we evaluated how the activity of CYP2D6, CYP3A4, CYP2C9, and CYP1A2 varied between normal weight, overweight, and morbidly obese individuals. These four CYPs were chosen due to their major involvement in the metabolism of drugs and their known pharmacogenetic polymorphisms. Moreover, we assessed the short- (1 month) and medium-term (6 month) impact of two bariatric surgery procedures on the activity of these CYP enzymes. Additionally, the previous parameters have been evaluated in the light of CYP polymorphisms.

## Results

### Effect of body mass index on CYP activities

We compared the values of CYP metabolic ratios (CYP2D6, CYP3A4, CYP2C9, and CYP1A2) among normal weight (*n* = 14), overweight (*n* = 14), and morbidly obese individuals (*n* = 24). The metabolic ratios of CYP2D6, CYP2C9, and CYP1A2 did not differ between the three body mass index (BMI) categories. However, CYP3A4 presented differences: morbidly obese individuals had a higher metabolic ratio (and thus a lower CYP3A4 activity) than normal weight ones (*p* = 0.022) (Table 1). CYP2C9 followed the same trend although the differences did not reach statistical significance. Overall, a high dispersion in the metabolic ratio values of all CYP isoforms was observed. This was more pronounced (up to 4 orders of magnitude) in the case of CYP2D6, revealing the importance of CYP2D6 genetic polymorphisms and subsequent inter-individual variability.

**Table 1:** Metabolic ratios of CYP enzymes in normal weight (*n* = 14), overweight (*n* = 14), and morbidly obese patients (*n* = 24) after a single oral administration of modified *Karolinksa cocktail* containing dextromethorphan (30 mg), losartan (25 mg), and caffeine (65 mg).

Metabolic ratio	Isoenzyme	Normal weight	Overweight	Morbid obese	Differences*
DXM/DX	CYP2D6	0.006 (0.004–0.008)	0.006 (0.005–0.056)	0.004 (0.002–0.024)	NS
DX/M3OL	CYP3A4	2.256 (1.959–3.067)	2.614 (2.117–3.722)	3.377 (2.876–4.076)	Normal weight < M. obese
Losartan/EXP-3174	CYP2C9	1.754 (1.303–3.017)	1.877 (1.691–2.292)	2.745 (1.458–4.125)	NS
Paraxanthine/Caffeine	CYP1A2	0.339 (0.277–0.463)	0.361 (0.272–0.450)	0.383 (0.261–0.575)	NS

Data are expressed as median (Q1, Q3).

Abbreviations: CYP: cytochrome P450, DXM: dextromethorphan, DX: dextrorphan, EXP-3174: the active metabolite of losartan, NS: not significant, M3OL: morphinan-3-ol (also known as 3-hydroxymorphinan).

*P < 0.05 using the log-transformed data.

**Figure 1:**
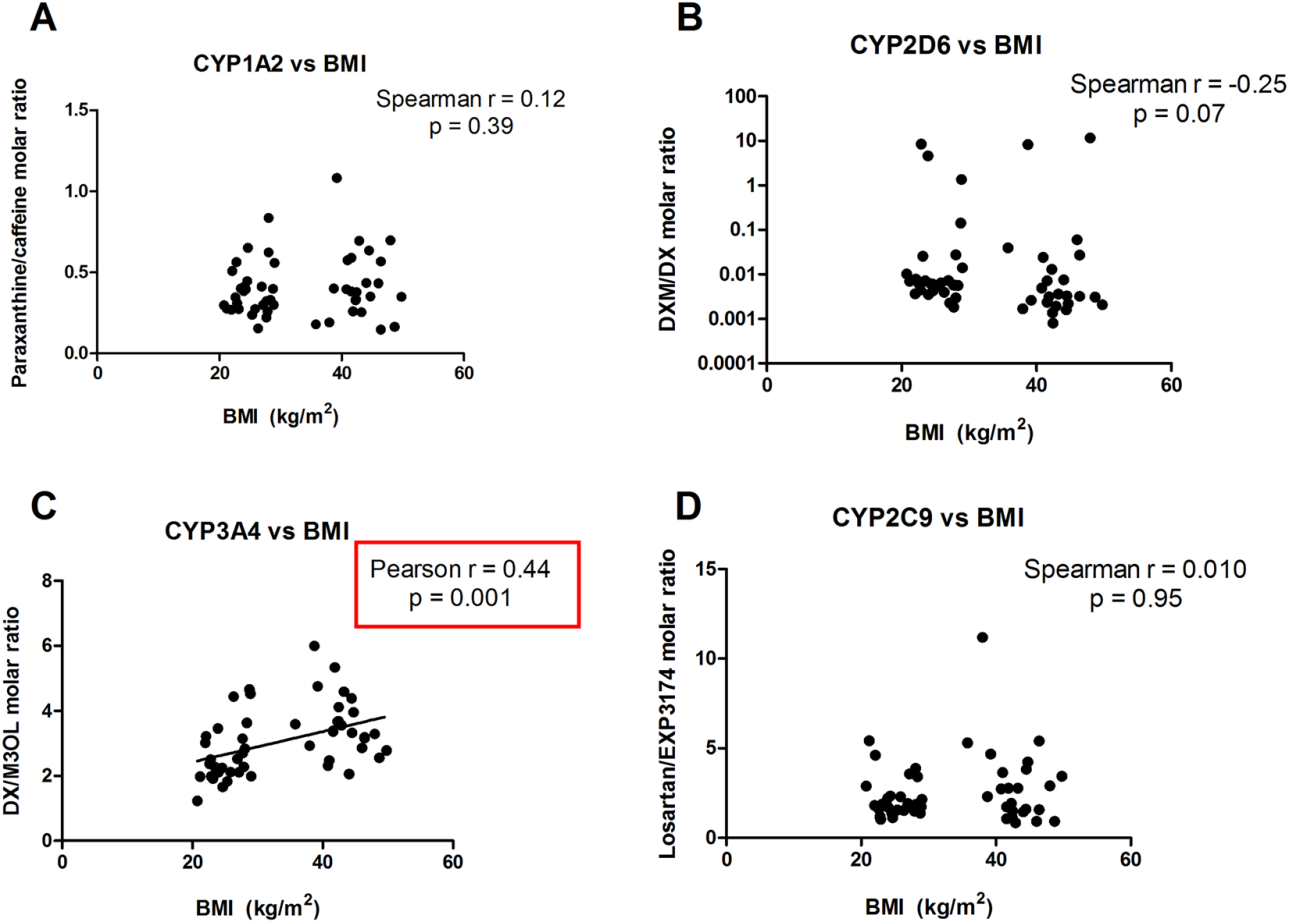
Correlation between BMI and CYP-mediated metabolic ratios at baseline. (**A**) Paraxanthine/caffeine molar ratio versus BMI; (**B**) Dextromethorphan/dextrorphan (DXM/DX) molar ratio versus BMI; (**C**) Dextrorphan/morphinan-3-ol (DX/M3OL) molar ratio versus BMI; (**D**) Losartan/EXP3174 molar ratio versus BMI.

Correlation analyses between CYP activities and BMI showed that CYP1A2, CYP2D6, and CYP2C9 activities did not correlate with BMI (Fig. 1). A negative correlation between CYP3A4 activity and BMI was, however, reported. This could be observed by the fact that the DX/M3OL ratio (CYP3A4-mediated substrate/product ratio) had a positive correlation with BMI (Pearson r = 0.44; p = 0.001), and indicated that as BMI increased, CYP3A4 activity decreased.

### Effect of two bariatric surgery techniques on CYP activities

We compared CYP metabolic ratios in morbidly obese individuals before (session 1), after 4 weeks (session 2), and after 6 months (session 3) of having undergone two different bariatric surgery techniques: laparoscopic sleeve gastrectomy (LSG) and laparoscopic Roux-en-Y gastric bypass (LRYGB) (Table 2).

**Table 2:** Phenotype indices of CYP enzymes in normal weight and morbid obese patients (n = 24) before (session 1), at 1 month (session 2), and 6 months (session 3) following sleeve gastrectomy (n = 10) or Roux-en-Y gastric bypass (n = 14).

Enzyme	Session 1 (Baseline)		Session 2 (1 month post-surgery)		Session 3 (6 months post-surgery)		Differences
	LRYGB	LSG	LRYGB	LSG	LRYGB	LSG	
CYP2D6	0.003 (0.002–0.009)	0.004 (0.002–4.172)	0.005 (0.004–0.010)	0.006 (0.002–2.324)	0.005 (0.003–0.015)	0.009 (0.003–5.079)	NS
CYP3A4	3.38 (3.17–3.68)	3.29 (2.78–4.59)	2.58 (2.31–3.32)	2.01 (1.62–2.53)	2.29 (2.05–2.58)	2.46 (1.91–2.74)	LRYGB S1 > S3 LSG S1 > S2,S3
CYP2C9	1.65 (1.24–3.55)	3.17 (1.77–4.56)	1.91 (1.13–3.50)	1.99 (1.45–3.03)	1.73 (1.28–3.33)	3.53 (1.76–3.92)	NS
CYP1A2	0.40 (0.33–0.58)	0.35 (0.19–0.61)	0.23 (0.19–0.33)	0.31 (0.14–0.66)	0.39 (0.29–0.57)	0.33 (0.27–0.78)	LRYGB S1 > S2 < S3

Data are expressed as median (Q1, Q3).

Abbreviations: CYP: cytochrome P450, LRYGB: Laparoscopic Roux-en-Y gastric bypass, LSG: laparoscopic sleeve gastrectomy, NS: non significant.

We did not observe any statistical difference in CYP2D6 metabolic ratios before and after each surgical technique, neither did we find any statistical difference in CYP2D6 metabolic ratios between the two procedures. We then combined the individuals from both interventions to assess the effect of bariatric surgery (irrespective of the technique employed). We observed that the CYP2D6 metabolic ratio tended to increase 6 months following surgery compared to basal values, although it did not reach statistical significance (p = 0.058).

**Figure 2:**
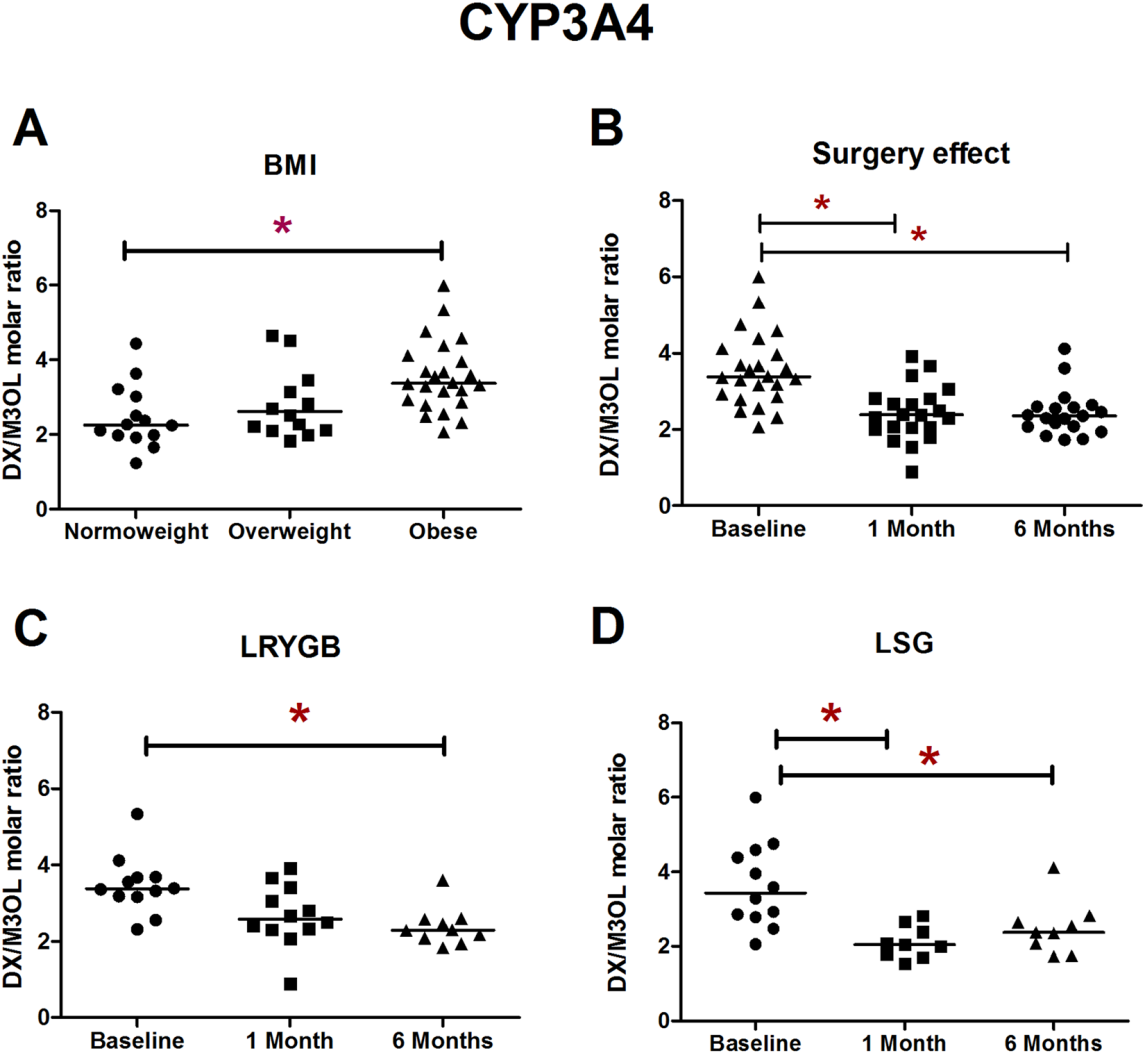
CYP3A4 phenotypic ratios (**A**) among normal weight, overweight, and morbid obese individuals; (**B**) in morbid obese patients prior to surgery (baseline), at 4 weeks post-surgery, and at 6 months; (**C**) after laparoscopic Roux-en-Y gastric bypass; (**D**) after laparoscopic sleeve gastrectomy. CYP3A4 phenotypic ratios were assessed by the urinary dextrorphan/morphinan-3-ol molar ratio (8 hour collection) after the intake of 30 mg of dextromethorphan. Abbreviations: BMI: body mass index, DX: dextrorphan, LRYGB: Laparoscopic Roux-en-Y gastric bypass, LSG: laparoscopic sleeve gastrectomy, M3OL: morphinan-3-ol (also known as 3-hydroxymorphinan).

The CYP3A4 activity augmented as a consequence of bariatric surgery (p < 0.001) as shown by the decreased CYP3A4 metabolic ratio over time. Whilst such effects were reported in both surgical techniques they occurred earlier in LSG (where statistical significance was achieved 4 weeks after surgery and maintained at 6 months) compared to LRYGB (where differences were observed 6 months after surgery) (Table 2 and Fig. 2).

No differences were found in CYP2C9 activity over time after any of the two studied techniques or between them.

In the case of CYP1A2, its activity temporarily decreased 1 month after LRYGB (*p* = 0.024) and recovered 6 months later. The same trend was observed after LSG although it did not reach statistical significance (Table 2).

### Influence of genotype

In order to evaluate the relevance of genotype on CYP activities, all the study participants were genotyped for CYP2D6, CYP3A4, CYP2C9, and CYP1A2 polymorphisms, and classified into groups according to their predicted phenotype and/or the number of functional alleles (in the case of CYP2C9 and CYP2D6) (see Supplementary Table 2). We then re-evaluated the effects of BMI and bariatric surgery on CYP2D6, CYP2C9, and CYP1A2 activities (this was not performed for CYP3A4 as there are no relevant polymorphisms).

### Effect of BMI and surgery on CYP activities considering genotype

**Figure 3:**
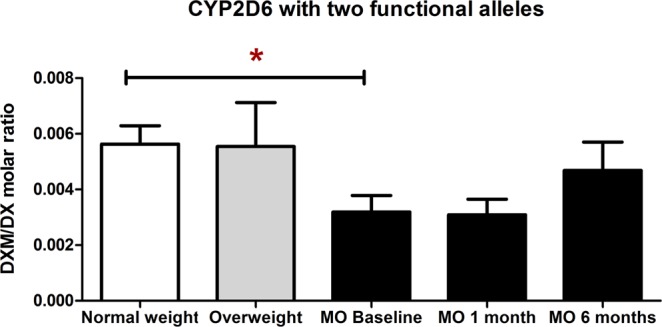
CYP2D6 phenotypic ratios in normal weight, overweight, and morbid obese subjects with 2 functional alleles before (baseline), at 1 month, and 6 months after bariatric surgery. CYP2D6 phenotypic ratios were assessed with the urinary dextromethorphan/dextrorphan molar ratio (8-hour collection). A lower dextromethorphan/dextrorphan molar ratio implies an increase in CYP2D6 metabolic activity, and viceversa.  Abbreviations: DXM: dextromethorphan, DX: dextrorphan, MO: morbidly obese.

CYP2D6 is a highly polymorphic gene with a great variety of allelic variants (>60). Amongst these, there are functional alleles, reduced function alleles, and non-functional alleles, which led to a wide range of activity (ranging from no metabolic activity to ultra-rapid activity). As explained in the first part of the manuscript, the metabolic ratios of CYP2D6 did not differ between the three BMI categories (Table 1). Additionally, we did not observe any statistical difference in CYP2D6 metabolic ratios before and after each surgical technique (Table 2). When we excluded the individuals that had either 0 or 1 functional alleles for CYP2D6, and we selected only those individuals that have 2 functional alleles for CYP2D6, this resulted in a lower sample size (n = 26) but with a fully functional CYP2D6 (which are the optimal conditions to study the activity of this enzyme). We observed that morbidly obese individuals carrying 2 functional alleles for CYP2D6 had a lower CYP2D6 metabolic ratio (i.e. higher enzymatic activity) compared to normal weight ones with 2 functional alleles (*p* = 0.035). Bariatric surgery tended to increase the values of the metabolic ratio although they did not reach statistical significance (Fig. 3).

No differences were found among groups (normal weight, overweight, and morbidly obese volunteers) for augmented and non-augmented CYP1A2 phenotypes. In the case of the CYP2C9 metabolic ratio, no differences were observed among groups with reduced, normal, and augmented CYP2C9 activity, or in individuals with 2 functional alleles for CYP2C9 (data not shown).

### Transient alterations at 1 month (session 2) which normalized at 6 months (session 3)

Another observation of this study is the fact that the CYP activities in some genotype subgroups were transiently altered 4 weeks after bariatric surgery and then normalized at 6 months. For instance, after excluding the two individuals who had a reduced CYP2C9 activity, the remaining morbidly obese individuals with a normal CYP2C9 phenotype who had undergone LSG presented a reduction in CYP2C9 metabolic ratio 4 weeks after surgery (*p* = 0.016) which normalized at 6 months. In a similar manner, as previously stated, the morbidly obese individuals who had undergone LRYGB displayed a reduction in CYP1A2 metabolic ratio 4 weeks after surgery (*p* = 0.024) which normalized at 6 months (Table 2). Further comments on these results are provided in the discussion.

## Discussion

In this study we report that (1) morbidly obese individuals have a lower CYP3A4 activity than normal-weight healthy controls; (2) the lower CYP3A4 activity found in morbid obese individuals is normalized after bariatric surgery; and (3) among those carrying two functional alleles for CYP2D6, morbidly obese individuals have a higher CYP2D6 activity than normal weight ones.

One of the major findings of this study is that CYP3A4 activity has a negative correlation with BMI. Indeed, CYP3A4 activity was lower in morbidly obese individuals compared to normal-weight controls. Morphinan-3-ol (M3OL) is the demethylated metabolite of dextrorphan. The urinary excretion of M3OL was 27% lower in morbidly obese individuals compared to the normal-weight group (8.23 vs 5.97 nmol), providing additional evidence that CYP3A4 activity is lesser in morbid obesity. Such results allow us to confirm the previous hypothesis that CYP3A4 activity may be decreased in obesity^19,22–24^. Indeed, previous studies have reported that in aged individuals, CYP3A4-catalyzed *N*-demethylation of erythromycin decreased as body weight increased^22^. The clearance of midazolam (a CYP3A4 substrate) was observed to be higher in obese adolescents than in morbidly obese adults, and the authors suggested that the greater clearance could be due to a lower CYP3A4 suppression^24^. The reduced CYP3A4 activity that we found in morbidly obese individuals could be a result of obesity comorbidities, as lower CYP3A4 protein expression and activity have been observed in non-alcoholic fatty liver disease and diabetes^25^.

Another major finding of this study is that CYP3A4 activity increased and then normalized following bariatric surgery. Normalization was observed 6 months after both surgical techniques and was already significant 4 weeks after LSG. Brill *et al*. studied the pharmacokinetics of midazolam in morbidly obese patients before and one year after bariatric surgery. They reported that midazolam clearance augmented after bariatric surgery and hypothesized that this could be due to a recovery in CYP3A activity^19^, which is compatible with our findings.

Since CYP3A4 is involved in the phase I metabolism of approximately 50% of all commercialized drugs^14^, the fact that CYP3A4 activity is decreased in morbid obesity and increased after surgery is relevant. In light of these results, further studies should confirm the influence of BMI on CYP3A4 activity and the potential need of dose adjustments of CYP3A4 substrates in order to maintain therapeutic action. Additionally, the results of this study indicate that special attention should be taken in patients undergoing bariatric surgery who are taking CYP3A4 substrates with a narrow therapeutic index (e.g. cyclosporine, tacrolimus, and sirolimus) as drug exposure will change^14^.

We did not observe any differences among the three BMI groups (normal weight, overweight, and morbidly obese) in the activity of the other CYPs studied (CYP2D6, CYP2C9, and CYP1A2). As genetic variants of CYP isoforms have been identified, and their impact on the resulting CYP activity established, we genotyped all the study participants and classified them accordingly. We then re-evaluated the influence of BMI and bariatric surgery on CYP activities taking into consideration genotype.

When genotype was considered, we found that morbidly obese individuals bearing 2 functional alleles for CYP2D6 had a higher CYP2D6 activity than normal-weight controls with 2 functional alleles for this enzyme. A more detailed analysis revealed that such an increase in CYP2D6 activity was due to the fact that morbidly obese individuals presented lower levels of dextromethorphan (around 50% of the amounts present in normal weight and overweight individuals, probably caused by a higher volume of distribution) but similar levels of dextrorphan (see Supplementary Fig. 1). Paulzen *et al*. employed a large naturalistic population sample (>2,200 individuals) to evaluate the levels of risperidone and its active metabolite 9-hydroxyrisperidone (whose hydroxylation is catalyzed by CYP2D6) in cachectic (<20 mg/kg^2^), normal weight, and obese individuals. The authors reported that BMI correlated positively with plasma concentrations of 9-hydroxyrisperidone^26^, a finding that is compatible with our observation that obese individuals (with 2 functional alleles for CYP2D6) have a higher CYP2D6 enzymatic activity than normal weight ones.

Another observation of our work is the temporary alteration in CYP activity observed at 4 weeks and normalized at 6 months in some subgroups. For instance, obese individuals with normal/intermediate CYP2C9 activity who had undergone an LSG had a reduction in CYP2C9 metabolic ratio 4 weeks after surgery (*p* = 0.016) which normalized at 6 months. In a similar manner, morbidly obese individuals with an augmented CYP1A2 activity who had undergone an LRYGB had a reduction in CYP1A2 metabolic ratio 4 weeks after surgery (*p* = 0.024) which normalized at 6 months. Such alterations are similar to our previously reported transient decrease in plasma concentrations of paraxanthine after bariatric surgery^21^. We hypothesize that these temporary alterations in CYP activities could be caused by the weight-loss-induced changes in hepatic function that take place after bariatric surgery and which preceded a significant functional recovery of the liver^27^.

The fact that we observed alterations in CYP3A4 and CYP2D6 activities is relevant since both enzymes metabolize more than half of the available marketed drugs. It is worth noting that both CYP3A4 and CYP2D6 activities are altered in morbid obesity although they follow different patterns (the first is decreased whereas the second is increased). This provides additional evidence that obesity-related alterations of CYP activities are isoenzyme-specific^23^.

The present study has some limitations. For instance, we evaluated the activity of 4 CYP isoenzymes, nevertheless, additional CYPs could be explored in the future (e.g. the polymorphic CYP2A6 and CYP2C19); we explored the effects of bariatric surgery up to 6 months but lack information regarding long-term CYP activities; only two bariatric surgery techniques were assessed, they are, however, the most commonly employed; all the morbid obese participants were women: among the 52 participants (28 controls/24 obese), there were 50 females and 2 males (the latter being in the control group), the results obtained, however, are not altered by the exclusion/inclusion of these two male subjects. On the other hand, the three major strengths of our study are the prospective and repeated measure design (in which each subject acts as the corresponding control, thus minimizing the influence of confounding variables and inter-individual variability); the inclusion of comparative groups (normal weight and overweight); and the fact that we genotyped all the participants for CYP2D6, CYP3A4, CYP2C9, and CYP1A2 using a DNA microarray that allows an accurate allele discrimination and screens for more than 34 genes and 85 allelic variants including 73 SNPs, 10 small insertions/deletions, and gene deletions and duplications^28^.

## Conclusion

In conclusion, in comparison with normal-weight controls, morbid obese individuals had a lower CYP3A4 activity, which normalized after bariatric surgery, and a higher CYP2D6 activity, which was only observed in those with two functional alleles for this isoenzyme. On the basis of our findings, dose adjustments might be necessary when treating morbidly obese patients with drugs that are substrates of CYP3A4. Clinicians should pay special attention when treating morbidly obese patients taking CYP3A4 substrates with a narrow therapeutic index (e.g. cyclosporine, tacrolimus, and sirolimus). Finally, further studies should evaluate whether the observed alterations in CYP3A4 activity will require dose adjustments for CYP3A4 substrates, especially following bariatric surgery.

## Methods

### Subjects

A prospective cohort study was conducted in 24 severely obese patients (aged 21–54 years) who met the 1991 bariatric surgery criteria of the National Institutes of Health^29^ and who were planning to undergo bariatric surgery at Hospital del Mar (Barcelona, Spain). Two different surgical procedures were considered: laparoscopic Roux-en-Y gastric bypass (LRYGB; *n* = 14) and laparoscopic sleeve gastrectomy (LSG; *n* = 10). Indications for the type of surgical procedure were based on clinical criteria and the consensus of the Bariatric Surgery Unit as previously described^30,31^. For comparative purposes, 14 overweight subjects (BMI 25–30 kg/m^2^), and 14 normal weight healthy control subjects (BMI < 25 kg/m^2^) were additionally included in the study. The socio-demographic baseline characteristics and basal clinical parameters of all the study participants have been described in a previous report^21^ and can be found in Supplementary Table 1.

### Study protocol

Participants were instructed not to consume ethanol, caffeine, and xanthine-related products (e.g., coffee, tea, colas, chocolate) for 2 days prior to the study sessions. This time window was chosen based on previous studies which reported that 16 hours of caffeine abstinence may be insufficient to avoid measuring methylxanthines remaining in the body, and suggested extending the abstinence period to 36 hours in future investigation^32^. In our study, we expanded this time period up to 48 hours to ensure that no remaining metabolites could interfere with the experiment. Subjects were excluded if they were taking medication known to induce or inhibit CYP enzymes or had a history of hypersensitivity to the study drugs.

After overnight fasting and baseline procedures, each subject received a modified *Karolinska cocktail*^32,33^ including 5 drugs: dextromethorphan (Romilar^®^, 30 mg), losartan (Cozaar^®^, 12.5 mg), omeprazole (Losec^®^, 20 mg), caffeine (65 mg), and paracetamol (Panadol^®^ Extra, containing 500 mg of paracetamol and 65 mg of caffeine). Dextromethorphan (30 mg) was self-administered the night before each session. Losartan (25 mg), paracetamol–caffeine (500 and 65 mg) were administered between 08:00 and 08:15, and omeprazole (20 mg) 1 hour later.

Blood samples (3 mL each) were taken at 0 and 4 h after oral caffeine administration. Urine was collected overnight after dextromethorphan administration (0–8 h), and during the experimental session following losartan/omeprazole/paracetamol/caffeine administration (0–8 h, from 08:00 until 16:00). Plasma samples were obtained from whole blood by centrifugation, urine samples were aliquoted, and both plasma and urine aliquots were stored at −20 °C until analysis.

This procedure was performed during one session in healthy volunteers (14 overweight and 14 normal weight control subjects) and three times in 24 morbidly obese patients: before surgery (session 1), at 4 weeks after surgery (session 2), and at 6 months (session 3).

All the morbid obese subjects were informed of the surgical risks and benefits, and signed their informed consent for the surgical procedure. In addition, they signed an informed consent for study participation.

### Surgical techniques

The LRYGB technique involved a 150-cm antecolic Roux limb with 25-mm circular pouch-jejunostomy and exclusion of 50 cm of the proximal jejunum. In LSG, the longitudinal resection of the stomach from the angle of His to approximately 5 cm proximal to the pylorus was performed using a 35 French bougie inserted along the lesser curvature. All operations were performed by the same team of surgeons.

### DNA extraction and genotyping

Genomic DNA was extracted from blood samples enriched with white cells (buffy coat) using the FlexiGene^TM^ DNA kit according to the manufacturer’s instructions (Qiagen, Spain). Allelic variants of CYP2D6, CYP3A4, CYP2C9, and CYP1A2 were analyzed with a DNA microarray (PHARMAchip^TM^; Progenika Biopharma SA, Spain), which includes 85 variants from 34 genes that encode five categories of proteins related to drug response^28^. The following allelic variants were analyzed: CYP2D6 (*1, *2, *3, *4, *5, *6, *7, *8, *9, *10, *11, *14 A, *14B, *15, *17, *18,*19, *20, *25, *29, *31,*35, *41, *1XN, *2XN, *4XN, *10XN, *17XN, *35XN, *41XN), CYP3A4 (*1, *1B), CYP2C9 (*1, *2, *3, *4, *5, *6), and CYP1A2 (*1, *1 F, *7, *11)^28^. For the subsequent analysis of CYP2D6 genotype, only the alleles *1, *2, and *35 were considered to be fully functional as described elsewhere^34^.

CYP2D6, CYP3A4, CYP2C9, and CYP1A2 genotypes were obtained for all the volunteers included in the study and each genotype was then classified according to the number of functional alleles. A detailed description of the participants’ genotypes for each studied CYP isoform can be found in Supplementary Table 2.

### Analytical methods

All chemicals were of the highest grade available. Dextromethorphan, dextrorphan, 3-methoxymorphinan, morphinan-3-ol, levallorphan (used as internal standard); caffeine, paraxanthine, and diphylline (used as internal standard); and losartan potassium were purchased from Sigma-Aldrich Quimica SA (Madrid, Spain). EXP-3174 was kindly provided by Merck Research Laboratories (New Jersey, USA).

Separation and quantification of dextromethorphan and its metabolites (3-methoxymorphinan, dextrorphan and morphinan-3-ol) in urine were performed by HPLC coupled with a fluorescence detector adapting a previously described method^35^. Briefly, a 2.5 mL aliquot of urine was spiked with 20 µL of internal standard solution (containing 100 µg/mL of levallorphan in methanol). Enzymatic hydrolysis of glucuronides was performed by the addition of 1.0 mL of *β*-glucuronidase HP-1 solution (pH 5; 0.1 M sodium citrate), followed by overnight (16 h) incubation in a water bath at 37 °C. Then 1 mL of ammonia/ammonium chloride buffer (pH 9.5) was added, the sample was mixed, centrifuged (5 min, 3500 rpm), and submitted to a solid-phase extraction using Bond Elut Certify columns (Varian, Palo Alto, CA, USA). Cartridges were conditioned with 2 mL of methanol and 2 mL of water. Samples were then loaded and the loaded cartridges washed with 2 mL of water, 1 mL of acetic acid 1 M, and 2 mL of methanol. Finally, elution of the retained compounds was performed with 2 mL of chloroform: propanol (80:20) containing 2% ammonia. After the evaporation of the solvent under a nitrogen stream (25 °C, 10–15 psi), they were reconstituted in 100 µL of a mixture of mobile phase, ultra-centrifuged at 10,000 rpm for 3 min, transferred to HPLC vials, and analyzed by HPLC coupled with a fluorescence detector using a previously described method.

Separation and quantification of plasma caffeine and paraxanthine concentrations were performed by HPLC coupled to an ultraviolet detector following a previously published methodology^36^. Briefly, a 150 µL aliquot of urine was spiked with 30 µL of NaOH 0.5 M, vortexed for 5 seconds and left at room temperature for 30 min. The mixture was acidified by adding 40 µL of HCl 0.5 M, vortexed for 5 seconds, and spiked with 30 µL of internal standard solution (containing 100 µg/mL of 9-methyluric acid). Then, 100 µL of a mixture containing dimethylformamide/ethyl acetate (30:70) and 800 µL of acetonitrile was subsequently added. The mixture was transferred to a microvial, centrifuged at 10,000 g for 5 minutes, and injected into the HPLC/UV system”.

Separation and quantification of urinary losartan and its metabolite E-3171 were carried out by HPLC coupled to a fluorescence detector as previously described without any modifications^37^.

### Metabolic ratios

CYP activities were estimated with previously described drug probes and validated metabolic ratios. For every batch analysis, a calibration curve was performed by adding known solutions of all analytes. Calibration curves with their corresponding slope (s), intercept, and coefficient of determination (r^2^) were calculated by weighting (1/x) least-squares linear regression of the peak area ratio (analyte/internal standard) versus the concentration of the standards. The obtained concentrations (expressed in µg/mL or ng/mL) were then converted into the corresponding molar concentrations (expressed in µmol/mL or nmol/mL). Finally, the molar ratios were calculated by dividing molar concentrations of the substrate by the product (or viceversa) as follows: CYP2D6 activity was estimated by the urinary (8-hour) dextromethorphan/dextrorphan molar ratio^38^. CYP3A4 activity was estimated by the urinary (8-hour) dextrorphan/morphinan-3-ol molar ratio^39,40^. CYP1A2 was estimated by the plasma (4-hour) paraxanthine/caffeine (17X/137X) molar ratio^41^. CYP2C9 was estimated as the urinary (8-hour) losartan/EXP-3174 molar ratio^42^. Note that CYP2D6, CYP3A4, and CYP2C9 metabolic ratios were calculated as substrate/product and thus a lower metabolic ratio implies a higher CYP activity and viceversa. However, in the case of CYP1A2 we decided to use the commonly employed paraxanthine/caffeine (calculated as product/substrate) and thus a lower metabolic ratio implies a lower CYP activity and viceversa. In addition, three of the CYP activities (CYP2D6, CYP3A4, and CYP2C9) were assessed by the analysis of urinary samples, and one CYP activity (CYP1A2) by measuring plasma samples. This decision was taken on the basis of previous reports describing that plasma, serum, and saliva are considered equally effective for measuring the paraxanthine/caffeine concentration ratio, whereas 30 different metabolic ratios have been proposed to measure CYP1A2 activity in urine after the administration of caffeine^43^. CYP2C19 activity evaluated by omeprazole was not analyzed and is not presented in this study.

### Statistical analysis

Given the fact that the interest variables were metabolic ratios, we log-transformed all the values before performing the statistical analyses. To compare the different weight categories (normal weight, overweight, and morbidly obese) with respect to metabolic ratios, one-way ANOVA models were used. Statistical significance was set at 0.05. A detailed description of the statistical analyses can be found in the Supplementary Material.

### Clinical trial registration

The study was approved by the local Research Ethical Committee (CEIC-Parc de Salut Mar, Barcelona, Spain) and Spanish Medicines Agency (EudraCT 2009-013156-72), and registered as a clinical trial in a public database (ClinicalTrials.gov NCT01086722).

### Ethical statement

All procedures performed in the study involving human participants were in accordance with the ethical standards of the institutional and/or national research committee and with the 1964 Helsinki declaration and its later amendments or comparable ethical standards.

### Consent statement

Informed consent was obtained from all individual participants included in the study.

## Supplementary information

Supplementary material.
