# Supplementary material for: Short- and medium-term impact of bariatric surgery on the activities of CYP2D6, CYP3A4, CYP2C9, and CYP1A2 in morbid obesity
Source: Sci Rep. 2019 Dec 31;9:20405. doi: 10.1038/s41598-019-57002-9 (PMC6938522; doi:10.1038/s41598-019-57002-9)
Supplement: Supplementary file 1 — Supplementary material. [file 41598_2019_57002_MOESM1_ESM.docx]

**Supplementary material**

**Short- and medium-term impact of bariatric surgery on the activities of CYP2D6, CYP3A4, CYP2C9, and CYP1A2 in morbid obesity**

Jose Rodríguez-Morató, Albert Goday, Klaus Langohr, Mitona Pujadas, Ester Civit, Clara Pérez-Mañá, Esther Papaseit, Jose Manuel Ramon, David Benaiges, Olga Castañer, Magí Farré_,_

Rafael de la Torre

***Statistical analysis***

Given the fact that the interest variables were metabolic ratios, we log-transformed all the values before performing the statistical analyses. To compare the different weight categories (normal weight, overweight, and morbidly obese) with respect to metabolic ratios, one-way ANOVA models were used. In order to evaluate if there were changes over time induced by surgery, repeated measures ANOVA models for the differences between session 1, session 2, and session 3 were fitted for all parameters including time as the only factor. In addition, to evaluate the differences between the surgical techniques, repeated measures ANOVA models for the differences between session 1, session 2, and session 3 were fitted for all parameters including time, type of surgery, and their interaction as factors. The computation of the simultaneous confidence intervals and adjusted p-values in order to guarantee a family-wise error rate of 0.05 is based on the multivariate t distribution of the vector of test statistics [[1](#_ENREF_1)]. The statistical analyses were carried out using the statistical software package R, version 3.4.3 (Vienna, Austria; <http://www.r-project.org/>). In particular, the packages “nlme” [[2](#_ENREF_2)] and “multcomp” [[1](#_ENREF_1)] were used to fit the repeated measures ANOVA models and to carry out the post-hoc comparisons, respectively. Statistical significance was set at 0.05. Pearson's and Spearman's

correlation coefficients (r) and P values were used to determine correlations between variables.

1. Hothorn T, Bretz F, Westfall P. Simultaneous inference in general parametric models. Biometrical Journal. 2008 Jun;50(3):346-363.

2. Pinheiro J BD, DebRoy S, Sarkar D and R Core Team nlme: Linear and Nonlinear Mixed Effects Models. R package version 3.1-137. 2018.

**Supplementary Table 1.**

Sociodemographic baseline characteristics and basal clinical parameters of the 24 patients, compared with normal weight and overweight volunteers. Data are expressed as mean ± SD. *Significant differences between the three subject groups. NS: Not significant

|  | Normal weight | Overweight | Obese | *Differences |
| --- | --- | --- | --- | --- |
| N | 14 | 14 | 24 | - |
| Age (years) | 32.2 ± 5.6 | 34.6 ± 6.6 | 41.4 ± 8.8 | *Normal, overweight < obese |
| Females | 12 | 14 | 24 | - |
| Males | 2 | 0 | 0 | - |
| Smokers | 36% | 29% | 38% | - |
| Height (cm) | 167 ± 9 | 161 ± 5 | 161 ± 9 | - |
| Weight (kg) | 64.4 ± 7.6 | 71.4 ± 5.4 | 111.6 ± 15.0 | *Normal < overweight < obese |
| BMI (kg/m^2^) | 23.0 ± 1.2 | 27.6 ± 1.1 | 43.1 ± 3.4 | *Normal < overweight < obese |
| SBP (mm Hg) | 107.3 ± 7.6 | 114.9 ± 9.9 | 124.3 ± 12.3 | *Normal < overweight < obese |
| DBP (mm Hg) | 66.6 ± 6.2 | 65.6 ± 6.9 | 68.8 ± 10.7 | NS |
| HR (bpm) | 61.4 ± 8.5 | 65.9 ± 7.0 | 68.4 ± 8.5 | *Normal < obese, overweight |

**Supplementary Table 2.**

Study participants’ genotypes and predicted phenotypes for CYP1A2, CYP2C9, CYP2D6 and CYP3A4.

|  |  | Caffeine | | Losartan | | Dextromethorphan | | | | |
| --- | --- | --- | --- | --- | --- | --- | --- | --- | --- | --- |
| Subject | BMI Category | CYP1A2 | | CYP2C9 | | CYP2D6 | | | CYP3A4 | |
|  |  | GENOTYPE | PREDICTED  PHENOTYPE | GENOTYPE | PREDICTED  PHENOTYPE | GENOTYPE | PREDICTED  PHENOTYPE | FUNCTIONAL  ALLELES* | GENOTYPE | PREDICTED  PHENOTYPE |
| Vol.001 | Normal weight | *1F/*1F | Augmented | *1/*1 | Normal | *1/*4 | Normal | 1 | *1/*1 | Normal |
| Vol.002 | Overweight | *1/*1F | Normal | *1/*1 | Normal | *1/*4 | Normal | 1 | *1/*1 | Normal |
| Vol.003 | Normal weight | *1F/*1F | Augmented | *1/*1 | Normal | *1/*2 | Normal | 2 | *1/*1 | Normal |
| Vol.004 | Normal weight | *1F/*1F | Augmented | *1/*1 | Normal | *1/*2 | Normal | 2 | *1/*1 | Normal |
| Vol.005 | Overweight | *1F/*1F | Augmented | *1/*1 | Normal | *2/*2 | Normal | 2 | *1/*1 | Normal |
| Vol.006 | Normal weight | *1/*1F | Normal | *1/*2 | Intermediate | *2/*2 | Normal | 2 | *1/*1 | Normal |
| Vol.007 | Overweight | *1F/*1F | Augmented | *1/*2 | Intermediate | *1/*4 | Normal | 1 | *1/*1 | Normal |
| Vol.008 | Normal weight | *1F/*1F | Augmented | *1/*2 | Intermediate | *2/*2 | Normal | 2 | *1/*1 | Normal |
| Vol.009 | Normal weight | *1/*1F | Normal | *1/*1 | Normal | *4/*4 | Poor | 0 | *1/*1 | Normal |
| Vol.010 | Normal weight | *1/*1 | Normal | *1/*1 | Normal | *1/*1 | Normal | 2 | *1/*1 | Normal |
| Vol.011 | Normal weight | *1F/*1F | Augmented | *1/*1 | Normal | *1/*4 | Normal | 1 | *1/*1 | Normal |
| Vol.012 | Normal weight | *1/*1F | Normal | *1/*1 | Normal | *1/*2 | Normal | 2 | *1/*1 | Normal |
| Vol.013 | Overweight | *1F/*1F | Augmented | *1/*2 | Intermediate | *1/*1 | Normal | 2 | *1/*1 | Normal |
| Vol.014 | Normal weight | *1F/*1F | Augmented | *1/*2 | Intermediate | *1/*9 | Normal | 1,5 | *1/*1 | Normal |
| Vol.015 | Normal weight | *1F/*1F | Augmented | *1/*1 | Normal | *1/*2 | Normal | 2 | *1/*1 | Normal |
| Vol.016 | Overweight | *1F/*1F | Augmented | *1/*1 | Normal | *1/*35 | Normal | 2 | *1/*1 | Normal |
| Vol.017 | Overweight | *1/*1F | Normal | *1/*1 | Normal | *4/*35 | Normal | 1 | *1/*1 | Normal |
| Vol.018 | Overweight | *1/*1F | Normal | *1/*1 | Normal | *5/*41 | Intermediate | 0,5 | *1/*1 | Normal |
| Vol.019 | Normal weight | *1/*1F | Normal | *1/*1 | Normal | *1/*1 | Normal | 2 | *1/*1 | Normal |
| Vol.020 | Overweight | *1/*1F | Normal | *1/*1 | Normal | *1/*4 | Normal | 1 | *1/*1 | Normal |
| Vol.021 | Normal weight | *1F/*1F | Augmented | *1/*1 | Normal | *1/*1 | Normal | 2 | *1/*1 | Normal |
| Vol.022 | Normal weight | *1F/*1F | Augmented | *1/*2 | Intermediate | *1/*2 | Normal | 2 | *1/*1 | Normal |
| Vol.023 | Overweight | *1F/*1F | Augmented | *1/*1 | Normal | *4/*5 | Poor | 0 | *1/*1 | Normal |
| Vol.024 | Overweight | *1/*1F | Normal | *1/*1 | Normal | *1/*1 | Normal | 2 | *1/*1 | Normal |
| Vol.025 | Overweight | *1/*1F | Normal | *1/*1 | Normal | *41/*41 | Intermediate | 1 | *1/*1 | Normal |
| Vol.026 | Overweight | *1/*1F | Normal | *1/*2 | Intermediate | *1/*2 | Normal | 2 | *1/*1 | Normal |
| Vol.027 | Overweight | *1/*1F | Normal | *1/*1 | Normal | *1XN/*1 | Extensive | 3 | *1/*1 | Normal |
| Vol.028 | Overweight | *1/*1F | Normal | *1/*1 | Normal | *1/*2 | Normal | 2 | *1/*1 | Normal |
| Vol.101 | Morbid obese | *1/*1F | Normal | *1/*2 | Intermediate | *2/*41 | Normal | 1,5 | *1/*1 | Normal |
| Vol.102 | Morbid obese | *1F/*1F | Augmented | *1/*1 | Normal | *1/*2 | Normal | 2 | *1/*1 | Normal |
| Vol.103 | Morbid obese | *1/*1F | Normal | *1/*2 | Intermediate | *1/*1 | Normal | 2 | *1/*1 | Normal |
| Vol.104 | Morbid obese | *1F/*1F | Augmented | *1/*1 | Normal | *2/*5 | Normal | 1 | *1/*1 | Normal |
| Vol.105 | Morbid obese | *1/*1 | Normal | *1/*2 | Intermediate | *2/*4 | Normal | 1 | *1/*1 | Normal |
| Vol.106 | Morbid obese | *1/*1F | Normal | *1/*2 | Intermediate | *1/*4 | Normal | 1 | *1/*1 | Normal |
| Vol.107 | Morbid obese | *1/*1F | Normal | *1/*1 | Normal | *1/*2 | Normal | 2 | *1/*1 | Normal |
| Vol.109 | Morbid obese | *1/*1F | Normal | *1/*1 | Normal | *1/*3 | Normal | 1 | *1/*1 | Normal |
| Vol.110 | Morbid obese | *1F/*1F | Augmented | *1/*1 | Normal | *2/*5 | Normal | 1 | *1/*1 | Normal |
| Vol.111 | Morbid obese | *1/*1F | Normal | *1/*3 | Reduced | *1/*1 | Normal | 2 | *1/*1 | Normal |
| Vol.112 | Morbid obese | *1/*1F | Normal | *1/*2 | Intermediate | *2/*2 | Normal | 2 | *1/*1 | Normal |
| Vol.113 | Morbid obese | *1/*1 | Normal | *1/*1 | Normal | *4/*5 | Poor | 0 | *1/*1 | Normal |
| Vol.114 | Morbid obese | *1/*1F | Normal | *1/*1 | Normal | *1/*1 | Normal | 2 | *1/*1 | Normal |
| Vol.115 | Morbid obese | *1F/*1F | Augmented | *1/*1 | Normal | *2/*10 | Normal | 1,5 | *1/*1 | Normal |
| Vol.116 | Morbid obese | *1/*1F | Normal | *1/*1 | Normal | *1/*41 | Normal | 1,5 | *1/*1B | Unknown |
| Vol.117 | Morbid obese | *1F/*1F | Augmented | *1/*1 | Normal | *1/*4 | Normal | 1 | *1/*1 | Normal |
| Vol.118 | Morbid obese | *1/*1F | Normal | *2/*3 | Reduced | *1/*4 | Normal | 1 | *1/*1B | Unknown |
| Vol.119 | Morbid obese | *1F/*1F | Augmented | *1/*2 | Intermediate | *2/*35 | Normal | 2 | *1/*1 | Normal |
| Vol.120 | Morbid obese | *1F/*1F | Augmented | *1/*1 | Normal | *2/*4 | Normal | 1 | *1/*1 | Normal |
| Vol.121 | Morbid obese | *1F/*1F | Augmented | *1/*2 | Intermediate | *4/*4 | Poor | 0 | *1/*1 | Normal |
| Vol.122 | Morbid obese | *1F/*1F | Augmented | *1/*1 | Normal | *1/*41 | Normal | 1,5 | *1/*1B | Unknown |
| Vol.123 | Morbid obese | *1F/*1F | Augmented | *2/*3 | Reduced | *1/*35 | Normal | 2 | *1/*1 | Normal |
| Vol.124 | Morbid obese | *1F/*1F | Augmented | *1/*1 | Normal | *2/*41 | Normal | 1,5 | *1/*1 | Normal |
| Vol.125 | Morbid obese | *1/*1F | Normal | *1/*1 | Normal | *1/*2 | Normal | 2 | *1/*1 | Normal |

*Based on Gaedigk A, Simon SD, Pearce RE, Bradford LD, Kennedy MJ, Leeder JS. The CYP2D6 Activity Score: Translating Genotype Information into a Qualitative Measure of Phenotype. Clinical Pharmacology & Therapeutics. 2008;83(2):234-242.

**Supplementary Figure 1.**

Urinary excretion (8-hour collection) of dextromethorphan (left) and dextrorphan (right) in study participants bearing 2 functional alleles for CYP2D6. The five experimental groups correspond to normal weight subjects, overweight subjects, morbid obese subjects before (baseline), 1 month, and 6 months after undergoing bariatric surgery.

1. Hothorn T, Bretz F, Westfall P. Simultaneous inference in general parametric models. Biometrical Journal. 2008 Jun;50(3):346-363. PubMed PMID: 18481363. Epub 2008/05/16. eng.

2. Pinheiro J BD, DebRoy S, Sarkar D and R Core Team nlme: Linear and Nonlinear Mixed Effects Models. R package version 3.1-137. 2018.
